# Clinical outcomes of pharmacologic versus non-pharmacologic interventions in postherpetic neuralgia: A retrospective cohort study

**DOI:** 10.1097/MD.0000000000043698

**Published:** 2025-08-08

**Authors:** Wei Yu, Jinzhao Lu

**Affiliations:** a Department of Pain, The Second Affiliated Hospital of Guangdong Medical University, Zhanjiang, Guangdong Province, China.

**Keywords:** epidemiology, pain management, pharmacological treatment, postherpetic neuralgia, quality of life, sleep quality

## Abstract

This study evaluates the comparative effectiveness and safety of pharmacologic and non-pharmacologic therapies in managing postherpetic neuralgia (PHN), aiming to refine clinical pain management approaches. A retrospective analysis was conducted on 60 patients diagnosed with PHN and treated between June 2022 and June 2024. Based on differences in prior treatment approaches, patients were matched and allocated into pharmacologic (n = 30) and non-pharmacologic (n = 30) treatment groups using propensity score matching to reduce baseline confounding. All participants were followed for 24 months. Primary endpoints included pain intensity (assessed via the visual analogue scale, VAS), health-related quality of life (measured by the SF-36 questionnaire), and sleep quality (evaluated using the Pittsburgh sleep quality index, PSQI). Adverse events were systematically recorded throughout the follow-up period. Both groups demonstrated statistically significant improvements in VAS, SF-36, and PSQI scores from baseline (*P* < .05). The pharmacologic group exhibited superior pain reduction at 12 months (mean VAS 4.5 ± 1.2 vs 5.0 ± 1.5; *P* < .05) and greater quality-of-life enhancement (SF-36 60.2 ± 7.9 vs 57.4 ± 8.4; *P* < .05). Sleep quality improvements favored pharmacologic therapy at 3 months (PSQI 12.0 ± 3.4 vs 13.4 ± 3.6; *P* < .05), though this difference attenuated by 12 months (*P* > .05). Adverse events were mild: pharmacologic interventions were associated with higher rates of dizziness (16.7%) and somnolence (10.0%), whereas non-pharmacologic methods primarily caused localized skin reactions (6.7%). Pharmacologic management demonstrated a faster short-term analgesic effect and was associated with improvements in quality of life in this study, while non-pharmacologic approaches appeared to offer potential benefits for long-term supportive care. These findings suggest that a combined therapeutic strategy could be a promising direction for optimizing PHN treatment.

## 1. Introduction

Postherpetic neuralgia (PHN) is one of the main complications of Herpes Zoster (HZ), characterized by severe pain that persists for more than a month after the rash has healed.^[1–3]^ The pathogenesis of PHN is complex and is closely related to the reactivation of the Varicella-Zoster Virus, as well as neuroinflammation and neuronal dysfunction.^[4,5]^ With the global trend of aging, the incidence of HZ and PHN has significantly increased, especially among the elderly. Studies have shown that more than 65% of HZ patients aged 60 and above may develop PHN, and the proportion for those aged 70 and above is as high as 75%.^[6,7]^ Therefore, PHN has become one of the important diseases affecting public health, imposing a heavy burden on patients, families, and the healthcare system.

In clinical practice, PHN is not only characterized by persistent pain but may also be accompanied by intermittent pain resembling electric shocks or knife cuts, as well as complex symptoms such as hyperesthesia or hypesthesia.^[8]^ This chronic pain state not only severely affects the quality of life of patients but may also lead to psychological issues such as depression, anxiety, and social disorders.^[9]^ Therefore, research on the epidemiological characteristics and pain management strategies of PHN is crucial. Although there have been many advances in the diagnosis and treatment of PHN internationally and domestically, due to the variety of treatment methods and individual differences in efficacy, how to formulate an optimized pain management plan remains a scientific question that needs to be resolved.

This study aims to analyze the epidemiological characteristics of PHN, including incidence, age and gender distribution, influencing factors, etc, based on the clinical data of 60 PHN patients collected from June 2022 to June 2024. It also systematically evaluates the effectiveness and safety of different pain management strategies, with the expectation of providing scientific and personalized treatment guidance for PHN patients. By exploring the synergistic effects of pharmacological treatment, non-pharmacological treatment, and psychological intervention, this study will provide new ideas for improving the quality of life of PHN patients. In addition, by revealing the risk factors and epidemic trends of PHN, this study will provide a basis for the prevention strategies and policy formulation of PHN, thereby effectively alleviating the social and economic burden brought by this complex disease.

## 2. Materials and methods

### 2.1. Study subjects

This study was approved by the Ethics Committee of the Second Affiliated Hospital of Guangdong Medical University. This study is a retrospective study that includes outpatients or inpatients diagnosed with postherpetic neuralgia (PHN) at a certain hospital from June 30, 2022, to June 30, 2024. All patients meet the International Association for the Study of Pain (IASP) diagnostic criteria for PHN, which is pain lasting for more than 1 month after the rash has healed.

To determine the sample size, we used the formula for comparing 2 means in a 2-group design. We aimed for a power of 80% and a significance level of 5% (α = 0.05). Based on a pilot study, we assumed a standard deviation of VAS scores to be 1.5 in both groups and a minimal clinically important difference of 1 point in VAS score. Using these parameters, the calculated sample size for each group was 28 patients. To account for a potential dropout rate of 10%, we increased the sample size to 30 patients per group, resulting in a total sample size of 60 patients. A total of 60 patients are planned to be recruited, and all patients have signed informed consent forms.

### 2.2. Sample size and grouping

The sample size was set at 60 cases, which was sufficient to meet the requirements for epidemiological analysis and pain management strategy evaluation based on the study design and follow-up duration. Patients were divided into 2 groups of 30 each using propensity score matching based on their prior treatment history, including demographic and clinical characteristics, to reduce selection bias. The 2 groups were as follows: pharmacological Treatment Group: received standard drug therapy, including pregabalin, gabapentin, or amitriptyline; non-pharmacological treatment group: received physical therapy (e.g., acupuncture, local heat application) or psychological intervention (e.g., cognitive-behavioral therapy).

### 2.3. Inclusion and exclusion criteria

Inclusion criteria: Age between 18 and 80 years, gender unrestricted; diagnosed with PHN by the pain department, with pain duration exceeding 1 month; able to understand the study content and sign an informed consent form; willing to accept follow-up and assessment on time.

Exclusion criteria: suffering from severe internal or surgical diseases, such as malignant tumors, liver or kidney dysfunction, etc; having mental illness or cognitive impairment, unable to cooperate with the study; having a clear history of allergy to any drugs or treatment methods used in the study; having participated in other related clinical trials within the recent month; pregnant or breastfeeding women.

### 2.4. Study indicators

Primary study indicators: pain intensity: assessed using the visual analogue scale (VAS).^[10]^ Quality of life: the SF-36 questionnaire is used to assess the improvement in patients’ quality of life.^[11]^

Secondary study indicators: adverse reactions: record the types and incidence of adverse reactions that occur during the treatment process. Sleep quality: the Pittsburgh sleep quality index (PSQI) is used to assess the improvement in patients’ sleep.^[12]^

### 2.5. Research methods

Baseline data collection: Collect basic information of patients, including age, gender, medical history, pain duration, and pain intensity, etc. Intervention measures: pharmacological treatment group: use first-line drugs such as pregabalin, gabapentin, or amitriptyline according to pain intensity and patient tolerance. Non-pharmacological treatment group: receive physical therapy 3 times a week or cognitive-behavioral therapy once a week. Follow-up: follow-up once a month, recording the patient’s pain intensity, quality of life, and adverse reaction conditions, with a follow-up period of 24 months. Data recording: use a unified data management system to enter all research data to ensure completeness and accuracy.

### 2.6. Statistical methods

1. Quantitative data were tested for normality using the Shapiro–Wilk test. Data conforming to normal distribution were expressed as mean ± standard deviation, and group comparisons were conducted using one-way analysis of variance (ANOVA). For non-normally distributed data, the Mann–Whitney *U* test was applied.
2. Categorical variables were expressed as frequency and percentage, and group differences were assessed using the chi-square (χ^2^) test or Fisher exact test, as appropriate.
3. Missing data were <5% for all key variables. To minimize bias, multiple imputation was performed using a fully conditional specification method under the assumption of missing at random.
4. All statistical analyses were conducted using SPSS version 26.0 (IBM Corp., Armonk), and a 2-sided *P*-value < .05 was considered statistically significant.

### 2.7. Ethical requirements

This study has been approved by the hospital’s ethics committee, and the research process strictly adheres to the Declaration of Helsinki and domestic ethical standards. All patients have signed informed consent forms, and their personal information will be strictly confidential and used only for the purposes of this study.

## 3. Results

### 3.1. Patient characteristics

A total of 60 patients with postherpetic neuralgia (PHN) were included in this study, with 30 in the pharmacological treatment group and 30 in the non-pharmacological treatment group. The average age of the patients was 65.1 ± 8.2 years, with 28 males (46.7%) and 32 females (53.3%). There were no statistically significant differences in age, gender, pain duration, and baseline VAS scores between the 2 groups (*P* > .05), indicating that the baseline was balanced and comparable (Table 1).

**Table 1:** Baseline characteristics of patients.

Characteristic	Drug treatment group (n = 30)	Nondrug treatment group (n = 30)	*F*-value/ χ^2^-value	*P*-value
Mean age (year)	65.2 ± 8.3	65.0 ± 8.1	0.012	.912
Male (n [%])	14 (46.7)	14 (46.7)	0.000	1.000
Female (n [%])	16 (53.3)	16 (53.3)	0.000	1.000
Pain duration (months)	7.3 ± 2.2	7.5 ± 2.4	0.033	.856
Baseline VAS score	8.3 ± 1.1	8.2 ± 1.0	0.012	.911

VAS = visual analogue scale.

### 3.2. Changes in pain intensity

After 3 months of treatment, the VAS scores of both groups significantly decreased compared to the baseline (*P* < .05), with the non-pharmacological treatment group’s VAS score being 6.5 ± 1.4 and the pharmacological treatment group’s being 5.7 ± 1.3, showing a statistically significant difference between the 2 groups (*P* < .05). At 12 months of treatment, the VAS score of the pharmacological treatment group further decreased to 4.5 ± 1.2, while the non-pharmacological treatment group decreased to 5.0 ± 1.5, with the difference still being statistically significant (*P* < .05) (Table 2).

**Table 2:** Changes in VAS scores between groups.

Time point	Drug treatment group	Nondrug treatment group	*F*-value	*P*-value
Baseline	8.3 ± 1.1	8.2 ± 1.0	0.012	.911
Month 3	5.7 ± 1.3	6.5 ± 1.4	4.658	<.05
Month 12	4.5 ± 1.2	5.0 ± 1.5	4.125	<.05

VAS = visual analogue scale.

### 3.3. Changes in quality of life

The SF-36 total scores of both groups significantly improved over the course of treatment (*P* < .05), with the pharmacological treatment group showing a greater improvement in physical health dimensions, while the non-pharmacological treatment group showed a more significant improvement in mental health dimensions. At 6 months of treatment, the SF-36 total score of the pharmacological treatment group increased from the baseline of 42.5 ± 8.6 to 60.2 ± 7.9, and the non-pharmacological treatment group increased from 41.8 ± 9.0 to 57.4 ± 8.4, with a statistically significant difference between the groups (*P* < .05) (Table 3).

**Table 3:** Changes in SF-36 total scores between groups.

Time point	Drug treatment group	Nondrug treatment group	*F*-value	*P*-value
Baseline	42.5 ± 8.6	41.8 ± 9.0	0.042	.842
Month 6	60.2 ± 7.9	57.4 ± 8.4	5.217	<.05

SF-36 = 36-item short form survey (quality of life questionnaire).

### 3.4. Changes in sleep quality

The PSQI scores of both groups significantly decreased after treatment (*P* < .05). At 3 months of treatment, the PSQI score of the pharmacological treatment group was 12.0 ± 3.4, which was significantly lower than the non-pharmacological treatment group’s score of 13.4 ± 3.6 (*P* < .05). At 12 months of treatment, the PSQI score of the pharmacological treatment group further decreased to 9.5 ± 2.8, while the non-pharmacological treatment group decreased to 10.2 ± 3.0, with no statistically significant difference between the 2 groups (*P* > .05) (Table 4).

**Table 4:** Changes in PSQI scores between groups.

Time point	Drug treatment group	Nondrug treatment group	*F*-value	*P*-value
Baseline	15.4 ± 3.2	15.6 ± 3.3	0.011	.915
Month 3	12.0 ± 3.4	13.4 ± 3.6	4.125	<.05
Month 12	9.5 ± 2.8	10.2 ± 3.0	0.996	.321

PSQI = Pittsburgh sleep quality index.

### 3.5. Adverse reactions

The common adverse reactions in the pharmacological treatment group were mild dizziness (5 cases, 16.7%) and sleepiness (3 cases, 10.0%), while the non-pharmacological treatment group experienced local skin redness and swelling (2 cases, 6.7%) and mild discomfort (1 case, 3.3%). No severe adverse reactions occurred in either group (Table 5).

**Table 5:** Adverse reactions in each group.

Adverse reaction	Drug treatment group (n [%])	Nondrug treatment group (n [%])	χ^2^-value	*P*-value
Dizziness	5 (16.7)	0 (0.0)	5.455	<.05
Drowsiness	3 (10.0)	0 (0.0)	2.941	.091
Local skin redness	0 (0.0)	2 (6.7)	2.308	.115

The above results indicate that the pharmacological treatment group was superior to the non-pharmacological treatment group in relieving pain, improving quality of life, and enhancing sleep quality, but the non-pharmacological treatment group had an advantage in improving mental health and had a good safety profile.

## 4. Discussion

This study compared the efficacy and safety of pharmacological and non-pharmacological treatments in patients with postherpetic neuralgia (PHN) and found that both treatment modalities significantly relieved pain, improved quality of life, and enhanced sleep quality. However, pharmacological treatment showed superior overall efficacy, providing important evidence for clinical treatment choices for PHN. The following discussion will delve into pain relief, quality-of-life improvement, sleep quality enhancement, and safety, and will also explore the significance and limitations of these findings in light of previous research.

### 4.1. Pain relief

Pain relief is the core objective of PHN treatment. This study demonstrated that both groups had significantly lower VAS scores at 3 and 12 months posttreatment compared to baseline, indicating that both pharmacological and non-pharmacological treatments effectively alleviated pain in PHN patients. However, the pharmacological treatment group had significantly lower VAS scores at both time points, suggesting a greater advantage in pain relief.

The pharmacological treatment group used calcium channel modulators such as pregabalin and gabapentin, which are recommended as first-line treatments in international guidelines.^[13,14]^ These drugs inhibit abnormal neuronal excitability, reducing the transmission of pain signals, and are particularly effective for managing continuous pain in PHN patients. The non-pharmacological treatment group primarily used physical therapies like acupuncture and heat application, as well as psychological interventions such as cognitive-behavioral therapy.^[15]^ These therapies have certain effects in relieving localized pain and adjusting pain perception but rely on activating endogenous analgesic pathways and enhancing psychological resilience, thus showing slower efficacy over time. Additionally, the effect of non-pharmacological treatments on pain may be more relieving mild to moderate pain and may be limited in patients with severe pain. This mechanism difference may be one of the reasons for the significant differences in efficacy between the 2 groups. Previous studies have also indicated that pharmacological treatment is the preferred option for pain relief in PHN.^[16,17]^ For example, a study by Shi et al showed that gabapentin significantly reduced pain scores in PHN patients and had a faster onset of action compared to placebo.^[18]^ The supplementary role of therapies such as acupuncture and heat application has also been verified, particularly in reducing local neural inflammation in patients. Therefore, a comprehensive treatment approach may be the direction for optimizing PHN management in the future.

### 4.2. Quality-of-life improvement

Improvement in quality of life is an important measure of PHN treatment outcomes. The results of this study showed that the SF-36 total scores of both groups significantly increased at 6 months posttreatment compared to baseline, indicating that both treatment modalities can improve the physical and mental health of patients to some extent. However, the pharmacological treatment group showed a significantly greater improvement in SF-36 total scores, particularly in the physical health dimension. Pharmacological treatment rapidly relieves pain, directly improving patients’ mobility and daily function, thereby increasing their physical health scores.^[19]^ Non-pharmacological treatments tend to improve patients’ psychological state through psychological support and overall regulation, which is more evident in the mental health dimension scores.^[20]^ However, due to the slower onset of non-pharmacological treatments and their limited effect on acute pain, the increase in physical health dimension scores is smaller. From a mechanistic perspective, PHN patients often suffer from emotional disorders, social withdrawal, and decreased life satisfaction due to long-term pain. Pharmacological treatment not only relieves pain but may also indirectly improve quality of life by improving sleep quality and emotional state. Non-pharmacological treatments help patients cope with pain through psychological intervention, which is particularly significant for improving anxiety and depressive symptoms in patients. In line with previous research, the role of psychological intervention in PHN patients is increasingly receiving attention. A study on cognitive-behavioral therapy showed that this therapy can significantly improve the psychological state and quality of life of chronic pain patients by helping them reshape their cognitive patterns of pain.^[21,22]^ Therefore, combining pharmacological and non-pharmacological treatments may be the best strategy to improve the quality of life of PHN patients.

### 4.3. Sleep quality enhancement

Sleep disturbances are common problems in PHN patients, mainly manifested as difficulty falling asleep, sleep maintenance disorders, and early awakening. The results of this study showed that the PSQI scores of both groups significantly decreased at 3 and 12 months posttreatment, indicating that both treatment modalities effectively improved patients’ sleep quality. However, the pharmacological treatment group had significantly better PSQI scores at 3 months, showing a greater advantage in early sleep quality improvement. Sleep disturbances in PHN patients are mainly related to chronic pain, and drugs such as pregabalin not only have analgesic effects but also certain sedative effects, significantly improving patients’ difficulty falling asleep and nocturnal awakening problems.^[23]^ Non-pharmacological treatments mainly improve sleep quality by relieving patients’ mental stress and reducing anxiety levels, thus taking a relatively longer time to take effect. This mechanism difference may explain the differences in PSQI scores between the 2 groups in the early follow-up. From the long-term follow-up results, at 12 months posttreatment, the difference in PSQI scores between the 2 groups was not statistically significant. This suggests that non-pharmacological treatments may gradually show efficacy similar to pharmacological treatments in long-term intervention. Considering the potential for tolerance and decreased compliance with pharmacological treatments, using non-pharmacological treatments as a supplementary means of long-term management is of great significance.^[24,25]^

### 4.4. Treatment safety

Treatment safety is an important consideration in clinical treatment. This study showed that neither group experienced severe adverse reactions, indicating that both pharmacological and non-pharmacological treatments have high safety. However, the pharmacological treatment group had a small number of mild adverse reactions such as dizziness (16.7%) and sleepiness (10.0%), which are consistent with the known side effects of pregabalin and gabapentin. The non-pharmacological treatment group had fewer adverse reactions, with only a small number of local skin redness and swelling, possibly related to improper heat application.^[15]^ Although the adverse reactions of pharmacological treatment are mild, they still need attention, especially for elderly patients, who should have their drug dosage adjusted based on individual differences. Non-pharmacological treatments have fewer adverse reactions, but standardized guidance for operations is needed to avoid discomfort caused by improper treatment procedures.^[15,26]^

### 4.5. Study significance and limitations

This study provides important clinical evidence for PHN pain management, highlighting the observed benefits of pharmacological treatment in alleviating pain, improving quality of life, and enhancing sleep quality. It also suggests the potential value of non-pharmacological interventions as supportive therapies in long-term management.

However, this study has several limitations. First, the sample size is relatively small (n = 60), with only 30 patients in each group. Although a power calculation was conducted during study design, the limited sample size restricts the statistical power, particularly in detecting smaller effect sizes and evaluating less frequent outcomes. As a result, the generalizability of findings is limited, and conclusions – especially regarding the differences in adverse reactions – should be interpreted with caution. The current sample does not provide sufficient power to draw definitive conclusions about the safety profile of each treatment modality. Second, the follow-up period of 24 months, although sufficient for mid-term outcome observation, may not be adequate for fully assessing the long-term sustainability, delayed adverse events, or recurrence of symptoms. Third, no stratified analyses were performed based on patient-level variables such as age, gender, comorbidities, or baseline symptom severity. These factors could influence treatment response and should be addressed in future studies.

Additionally, there were notable differences in the type and frequency of interventions between the pharmacologic and non-pharmacologic groups. The pharmacologic group primarily received standardized medications (e.g., pregabalin, gabapentin), while the non-pharmacologic group included diverse approaches such as acupuncture and cognitive-behavioral therapy, each with varying intensity and session frequency. These inconsistencies may have introduced potential confounding and reduced internal comparability between groups. Future studies should aim to standardize treatment components or control for intervention intensity to improve methodological consistency.

### 4.6. Clinical implications

The results of this study suggest that for PHN patients, pharmacological treatment should be the first choice, and non-pharmacological treatment can be used as a supplementary means, especially for patients with poor response to pharmacological treatment or obvious adverse reactions. Combined treatment may be an important direction for optimizing PHN management in the future. Clinical practice should focus on individualized treatment, formulating comprehensive management plans based on patients’ pain characteristics, psychological state, and living needs, thereby maximizing pain relief and improving quality of life.

In summary, this study provides preliminary evidence supporting the potential benefits of both pharmacological and non-pharmacological treatments for PHN. Pharmacological interventions demonstrated a faster analgesic effect in the early phase, while non-pharmacological approaches may offer complementary advantages in long-term symptom management. These findings suggest that an integrated therapeutic strategy could contribute to improved clinical outcomes and enhanced quality of life for patients with PHN.

## Author contributions

**Conceptualization:** Wei Yu, Jinzhao Lu.

**Data curation:** Wei Yu, Jinzhao Lu.

**Formal analysis:** Wei Yu, Jinzhao Lu.

**Investigation:** Wei Yu, Jinzhao Lu.

**Methodology:** Wei Yu, Jinzhao Lu.

**Supervision:** Wei Yu, Jinzhao Lu.

**Validation:** Wei Yu, Jinzhao Lu.

**Visualization:** Wei Yu, Jinzhao Lu.

**Writing – original draft:** Wei Yu, Jinzhao Lu.

**Writing –review & editing:** Wei Yu, Jinzhao Lu.
